# Wide Local Excision of Perianal Squamous Cell Carcinoma Associated With Low-Risk Human Papillomavirus (HPV) Subtype

**DOI:** 10.7759/cureus.82335

**Published:** 2025-04-15

**Authors:** Jordan Roy, Bjarne Faraon, Michael Polcino

**Affiliations:** 1 General Surgery, St. Barnabas Hospital, Bronx, USA; 2 Surgery, City University of New York (CUNY) School of Medicine, New York, USA; 3 Colorectal Surgery, St. Barnabas Hospital, Bronx, USA

**Keywords:** anal, cancer, excision, hpv, invasive squamous cell carcinoma, perianal

## Abstract

There is a strong association between the incidence of perianal cancer and high-risk human papillomavirus (HPV) subtypes, most notably HPV-16. In contrast, low-risk subtypes, such as HPV-6 and HPV-11, are commonly implicated in the development of condyloma acuminatum or anogenital warts. We present the case of a 40-year-old male patient with a past medical history of hypertension and congenital HIV, which was controlled on highly active antiretroviral therapy (HAART), who presented with a chief complaint of pain at the site of a large fungating perianal mass. A tissue biopsy taken during laparoscopic descending loop colostomy, created to divert stool away from the mass, showed squamous cell carcinoma (SCC) with positive HPV-6 and HPV-11 markers. Following multi-week nutritional optimization, wide local excision (LE) of the tumor followed by immediate plastic reconstruction and closure was performed. The final pathology of the specimen disclosed it as a T4 18 x 17 x 5 cm invasive SCC with moderate differentiation and no lymphovascular invasion. While the association between high-risk HPV and perianal cancer has been well studied and characterized, evidence regarding the development of perianal cancer in the presence of low-risk HPV subtypes is limited to isolated case reports. As a result, there are currently no established guidelines for annual perianal cancer screening in patients with either known low- or high-risk HPV. However, screening does exist for postoperative recurrence of anal and perianal SCC. Due to the increased risk of developing perianal cancer from HPV infection, particularly the high-risk subtype, individualized screening should be emphasized. Overall, further research is needed to develop a standardized protocol that can potentially help reduce the incidence of perianal cancer in high-risk populations with either low-risk or high-risk HPV.

## Introduction

Giant condyloma acuminatum or Buschke-Lowenstein tumor is a sexually transmitted disease caused by infection with human papillomavirus (HPV) subtypes 6 and 11 [1]. HPV infection manifests as hypertrophy of the infected tissue, characterized by thick, discrete lesions commonly referred to as warts [2]. HPV possesses viral oncoproteins, E6 and E7, that drive cells toward oncogenesis. E6 targets p53, an important growth suppressor, while E7 targets pRB [3]. The dysregulation of these growth suppressor proteins leads to subsequently uncontrolled proliferation of infected cells.

According to the latest report of the Centers for Disease Control and Prevention (CDC) on HPV and its epidemiology, the prevalence of any of the HPV genital subtypes was 45.2% among US men aged 18-59 years and 39.9% among US women in the same age range [4]. HPV is responsible for mucocutaneous and anogenital lesions that can progress to cancer and is implicated in the pathogenesis of 91% of cervical and anal cancers, 69% of vulvar cancers, 75% of vaginal cancers, 63% of penile cancers, and 70% of oropharyngeal cancers [4]. There is an estimated 27,000 new cases of anal cancer each year attributable to HPV-associated infection [5]. Risk factors include immunosuppression and a history of venereal disease. HIV-positive men who have a CD4 cell count < 200 cells/mm^3^ have a threefold increase in the transformation rate of normal epithelium to anal squamous intraepithelial lesions [6].

It is important to note that among the numerous HPV subtypes, this article focuses on those classified as carcinogenic or probably carcinogenic (HPV subtypes 16, 18, 31, 33, 35, 39, 45, 51, 52, 56, 58, 59, and 68). According to a meta-analysis aimed at identifying the relationship between these high-risk HPV subtypes and the development of anal cancers, HPV-16 has been shown to be the most carcinogenic subtype. Among all identified anal cancers, the HPV-16 subtype was present in over 85% of cases [7]. With regard to perianal skin cancers specifically, there is a paucity of data; however, the HPV-16 subtype has also been suggested to be the most prevalent and subsequently carcinogenic subtype. An analysis by Bjørge et al. [8] of Finnish and Norwegian patient registries found that over half of perianal cancers, histologically identified as squamous cell carcinoma (SCC), were seropositive for HPV subtypes 16, 18, 33, and 73, with HPV-16 being the most prevalent. Moreover, HPV-16 infection carries the highest carcinogenic potential in the development of anal and perianal cancers. 

The anal canal, which is about 4-5 cm long, begins from the lower rectal mucosa and includes the area where rectal mucosa transitions to the modified squamous epithelium of the anal canal, along with the dentate line. It extends to the point where the skin becomes keratinized at the anal verge. In essence, anal canal cancer encompasses any abnormality that cannot be fully observed through external examination alone. Perianal cancer encompasses lesions visible during external examination, requiring the separation of the buttocks to a distance of up to 5 cm from the anus. Lesions beyond this 5 cm radius are classified as skin cancers [9].

Treatment with excision alone with a 1 cm margin is only reserved for well-differentiated T1 N0 M0 perianal tumors [10]. After excision with negative margins, monitoring for five years with digital rectal exam, inguinal exam, and anoscopy every 3-6 months is recommended [10]. Positive margins require re-excision and chemoradiotherapy (CRT). All other perianal tumors T2 or greater +/- poorly differentiated T1 N0 and tumors of the anal canal require CRT with mitomycin and 5-fluorouracil [9].

Although HPV subtypes 6 and 11 are considered low risk, as they tend to cause warts rather than cancer [11], we present a case of invasive perianal SCC arising from a giant condyloma acuminatum positive for HPV-6 and HPV-11.

## Case presentation

**Figure 1:**
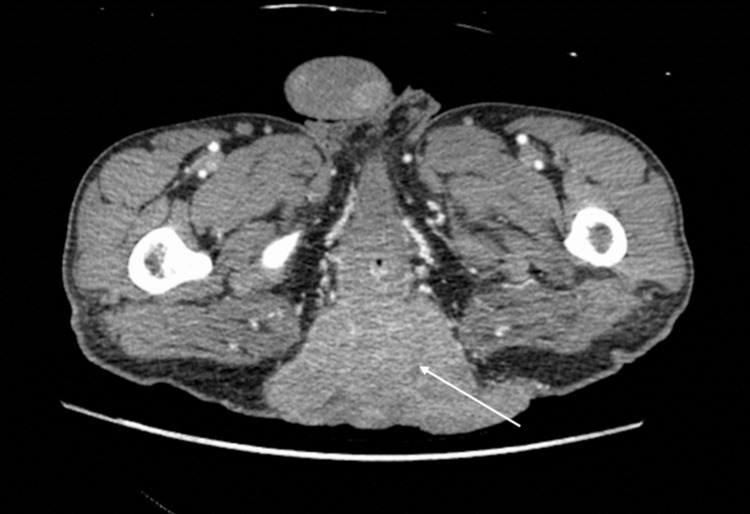
Abdominal and pelvic CT demonstrating a large fungating perianal mass abutting the anus.

The patient is a 40-year-old man with a medical history of hypertension and congenital HIV, well controlled on HAART, who presented with a chief complaint of pain at the site of a large fungating perianal mass. An initial physical exam revealed frank purulent drainage from the mass with a leukocytosis of 25.7. A CT scan was performed to further characterize the mass and assess for metastatic disease (Figure 1). No evidence of metastasis was observed in the chest, abdomen, or pelvis. The patient reported that the mass had initially been smaller and was previously resected; however, it recurred. He was lost to follow-up with his initial surgeon and did not undergo appropriate surveillance for recurrence. The patient was started on IV antibiotics and, once stabilized, underwent an index operation consisting of an examination under anesthesia with tissue biopsy and laparoscopic creation of a descending loop colostomy to divert stool from the affected area. Histopathological analysis confirmed the diagnosis: SCC positive for HPV-6 and HPV-11 markers.

After the index operation, the decision was made to perform a complete colonoscopy through both the proximal and distal loops of the colostomy. This would not only inform of any other masses in the colon but would also help elucidate if the mass involved the anal canal or rectum. Involvement of these structures would shift the diagnosis from perianal SCC to anal canal SCC, which is more appropriately treated with chemoradiation (Nigro protocol) rather than surgical resection [12]. The colonoscopy revealed no additional lesions and showed no evidence of invasion into the anal canal.

**Figure 2:**
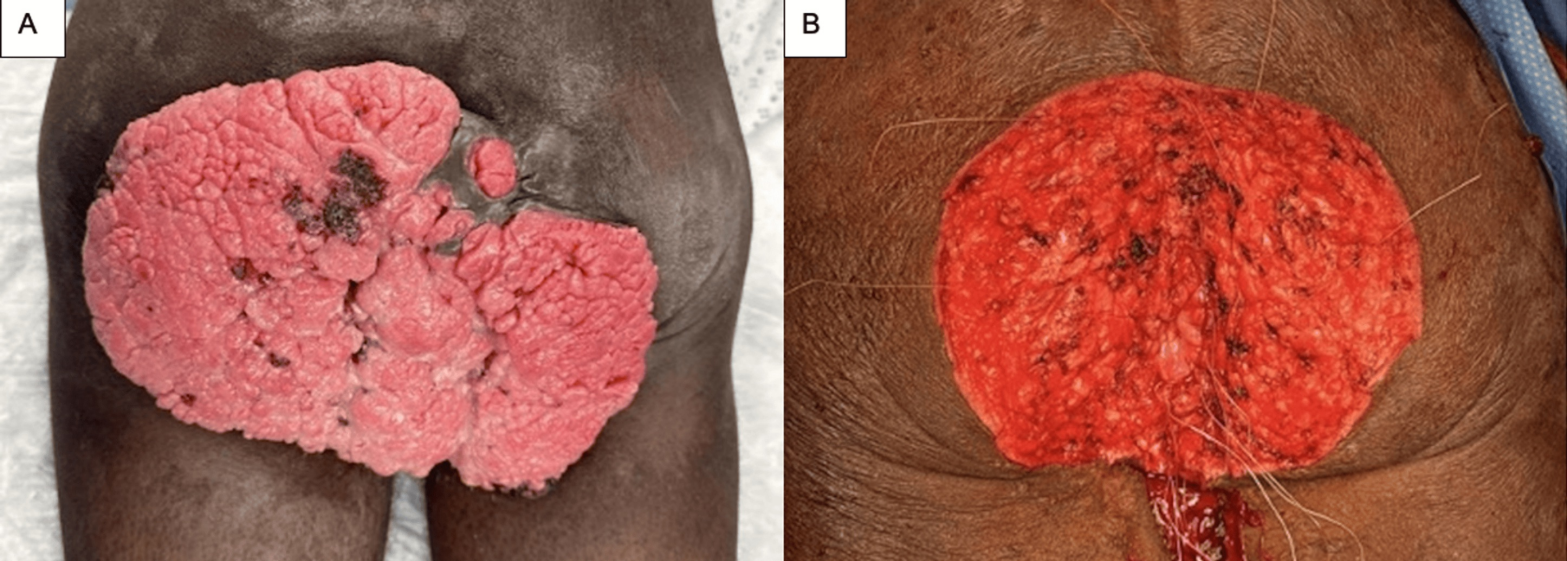
Preoperative (A) and postoperative (B) photographs of wide local excision of perianal SCC tumor with preservation of the anus. SCC: squamous cell carcinoma.

The patient was then nutritionally optimized to support healing of the anticipated excision site. He was finally taken to the operating room for a wide local excision (LE) of the tumor. The patient was placed in a prone jackknife position, and a skin incision was made around the tumor, which was then dissected through subcutaneous tissue and fat to the level of the gluteus muscle. Care was taken to avoid and control bleeding while continuing resection. Finally, once the mass was excised, the anus was reapproximated to the sphincter complex using sutures (Figures 2A, 2B). The levator muscles were preserved, and immediate reconstruction was then performed by the plastic surgery team, which the patient tolerated well.

The final pathology revealed an 18 x 17 x 5 cm moderately differentiated invasive SCC without lymphovascular invasion. The tumor was staged as T4. The patient did well postoperatively and was discharged on postoperative day 9.

**Figure 3:**
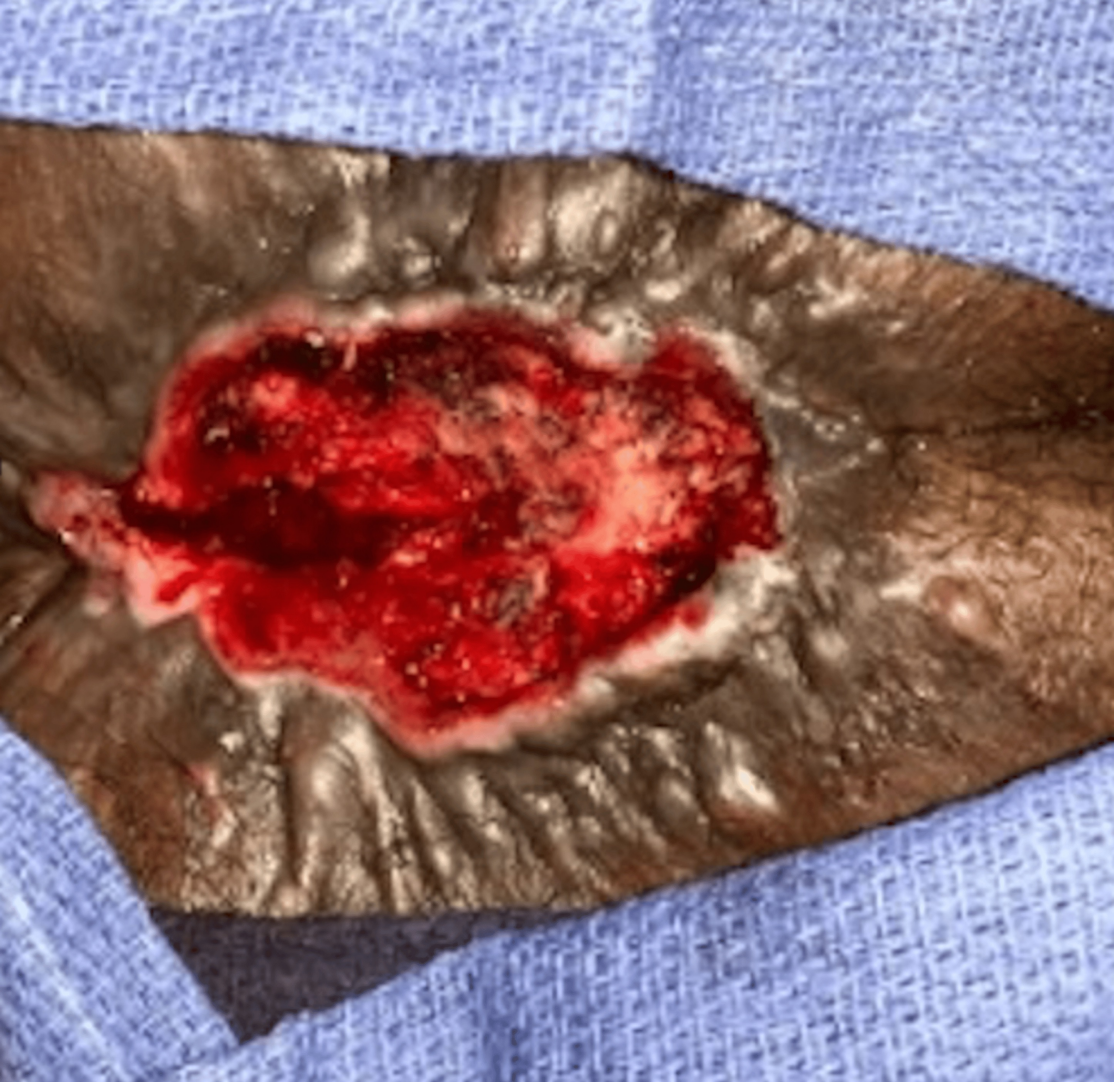
Thirty-day postoperative photograph of wide local excision of perianal SCC tumor with preservation of the anus, minor dehiscence of closure but with overall healthy granulation tissue. SCC: squamous cell carcinoma.

Figure 3 depicts the surgical site 30 days postoperatively, demonstrating healing following wide LE of the perianal SCC with preservation of the anus.

## Discussion

This case presents a patient with T4 invasive perianal SCC, caused by low-risk HPV subtypes 6 and 11. It is estimated that 90% of cervical and anal cancers are due to infections from high-risk HPV subtypes 16 and 18 [13]. On the other hand, developing anal or perianal cancer from low-risk HPV subtypes is quite rare. One study analyzing the etiology in a population of 81 cases of anal cancer found that the absolute frequency of HPV-6 and HPV-11, respectively, detected in anal cancer was less than five. Comparatively, the absolute frequency of HPV-16 detected in a single infection and multiple infections in anal cancer was greater than 45 [14]. Another study found an association between mono-infection by HPV-6 and progression to high-grade squamous intraepithelial lesion (HSIL) [15]. Furthermore, individual case reports have reported chronic mono-infection by HPV genotypes 6, 11, 42, 44, or 70 to be the cause of a small number of perianal cancers [16]. Overall, research has shown that while high-risk HPV has a significantly stronger association with perianal and anal canal cancers, there still may be a potential relationship with low-risk HPV subtypes as well.

Currently, there are no uniform established guidelines for annual perianal cancer screening in patients with known low- or high-risk HPV. However, the International Anal Neoplasia Society has recommended that individuals aged ≥45 years with a history of cervical/vaginal HSIL or cancer, perianal warts, persistent (>1 year) cervical HPV-16, or autoimmune conditions could be considered for screening with high-resolution anoscopy, anal cytology, high-risk HPV testing, and hrHPV cytology co-testing [17]. There does, however, exist screening for postoperative recurrence of anal SCC. This algorithm recommends surveillance for five years. A digital rectal exam and inguinal node exam every 3-6 months are recommended for five years. Anoscopy is recommended every 6-12 months for three years. CT scans of the chest, abdomen, and pelvis are recommended every 12 months for three years [9]. Furthermore, this algorithm recommends treatment with groin dissection in those with positive lymph nodes and salvage abdominoperineal resection with or without extended resection in those with locoregional cancer recurrence. For patients with metastatic recurrence, systemic therapy is advised.

HPV prevalence is highest in young adults, with prevalence increasing with age from 14 to 24 years and peaking between the ages of 25 and 29. Furthermore, Hispanic Black adults have the highest prevalence of genital HPV, while non-Hispanic Asian adults have the lowest. On the other hand, White men and women have a higher incidence of HPV-associated cancers while Asian/Pacific Islander men and women have the lowest [18,19]. Specific populations at increased risk for developing HPV-related anal or perianal cancer include men who have sex with men; HIV-positive individuals; women with a history of cervical, vulvar, or vaginal cancer; and elderly people [20]. Therefore, given the increased risk of developing cervical and anal cancers from infection with HPV, particularly the high-risk subtype, individualized screening should be emphasized in patient populations that are stratified to be “high risk” based on prior research. Given the lack of standardized guidelines for anorectal cancer screening in HPV-positive patients, further research is needed to develop a standardized screening protocol that can potentially help reduce morbidity and mortality of cancer in high-risk populations. Currently, the standard of care for early-stage perianal cancer is wide LE. LE is preferred due to the ability to preserve anal function in contrast to radical resection, which carries an additional risk for urinary and erectile dysfunctions. Generally, the practice of LE is reserved for lesions that are stage T2 or lesser, without any evidence of nodal involvement or metastases. One study examining the effectiveness of LE for early-stage perianal cancer found a five-year disease-free survival rate of approximately 71%, which improved to 83% with adjuvant chemoradiotherapy. Despite this five-year disease-free survival rate, LE is still inferior with regard to overall survival as compared to radical resection. Therefore, careful evaluation of tumor invasion is necessary to determine the appropriateness of LE, and patients should undergo routine follow-up to monitor for recurrence. As suggested by Wietfeldt, an anorectal and nodal examination should be performed every three months for two years following surgery and subsequently biannually until year 5. In the setting of advanced tumors, particularly T3 and T4 lesions, treatments with chemotherapy, external beam radiation therapy (ERBT), and interstitial implant therapy have been demonstrated to be highly effective in obtaining locoregional control. In our patient, despite his advanced T4 lesion, LE of the tumor proved to be effective. Strict adherence to screening for recurrence was emphasized due to the inherent risk of recurrence of the wide LE technique.

## Conclusions

As demonstrated by this case, the association between low-risk HPV and perianal cancer, while rare, is possible and may present in isolated cases. In cases like our patient, timely management is often critical. The absence of a standardized protocol for such presentations can delay treatment and contribute to poor outcomes, including increased morbidity and mortality. In this case, treatment was tailored to the patient’s clinical presentation and intraoperative findings, resulting in a favorable postoperative course. Nonetheless, the patient may have benefitted from closer surveillance following his initial surgery, as he was lost to follow-up until the mass recurred and progressed to a T4 invasive SCC. Fortunately, he is now clinically stable, gaining weight, and has undergone successful ostomy reversal. This case highlights the importance of both individualized management and the need for standardized protocols. We hope that our report, along with further research, will contribute to the development of guidelines for the screening and treatment of HPV-associated anal and perianal cancers.
